# A non-invasive method to genotype cephalopod sex by quantitative PCR

**DOI:** 10.1101/2025.10.28.685099

**Published:** 2025-10-29

**Authors:** Frederick A. Rubino, Gabrielle C. Coffing, Connor J. Gibbons, Scott T. Small, Thomas Desvignes, Jeffery Pessutti, Ann M. Petersen, Alexander Arkhipkin, Zhanna Shcherbich, John H. Postlethwait, Andrew D. Kern, Tessa G. Montague

**Affiliations:** 1 The Mortimer B. Zuckerman Mind Brain Behavior Institute, Department of Neuroscience, Columbia University, New York, NY, USA; 2 Department of Biology, Whitehead Institute, Massachusetts Institute of Technology, Cambridge, MA, USA; 3 These authors contributed equally; 4 Institute of Ecology and Evolution, University of Oregon, Eugene, OR, USA; 5 Department of Biology, University of Alabama at Birmingham, Birmingham, AL, USA; 6 Institute of Neuroscience, University of Oregon, Eugene, OR, USA; 7 National Oceanographic and Atmospheric Administration, NOAA Fisheries, Northeast Fisheries Science Center, JJ Howard Marine Laboratory, New Jersey, USA; 8 Gloucester Marine Genomics Institute, MA, USA; 9 Fisheries New Zealand, Wellington, New Zealand; 10 Falkland Islands Fisheries Department, Stanley, Falkland Islands.; 11 Howard Hughes Medical Institute, Columbia University, New York, NY, USA; 12 Lead contact

**Keywords:** Cephalopod, cuttlefish, octopus, squid, genotype, sex determination, Z chromosome, aquaculture, karyotype

## Abstract

Coleoid cephalopods (cuttlefish, octopus, and squid) are emerging model organisms in neuroscience, development, and evolutionary biology, and are of major economic importance in global fisheries. However, they are notoriously difficult and expensive to culture. The ability to determine sex early in development would enable more efficient and sustainable population management in both lab and wild settings. Here, we present a non-invasive method to genotype the sex of dwarf cuttlefish (*Ascarosepion bandense*) as young as three hours post-hatching using a skin swab and quantitative PCR assay, which detects a two-fold dosage difference between ZZ and Z0 sex chromosomes of males and females, respectively. Furthermore, we designed and validated primers for four additional cephalopod research species with assembled genomes (*Octopus bimaculoides, Sepia officinalis, Euprymna berryi*, *Doryteuthis pealeii*), and for a wild-caught species of economic value (*Illex illecebrosus*) for which we generated low-coverage whole genome sequencing data. This sex-genotyping method enables accurate sex determination from hatchlings to adults across cephalopods, independent of genome quality or availability.

## Introduction

The coleoid cephalopods – octopus, cuttlefish and squid – are a group of marine mollusks with a striking array of biological adaptations, including dynamic camouflage, regenerative limbs, the largest brain-to-body-ratio of the invertebrates, tactile chemosensation (“taste by touch”), extensive RNA editing, and skin-based social communication^1–6^. The last common ancestor of the cephalopods and vertebrates lived ~600 million years ago^7^, prior to the emergence of centralized nervous systems. Since then, cephalopods have evolved the largest and most complex brains among invertebrates – capable of learning, memory, problem-solving, and adaptive camouflage^8–13^. There is growing interest in the scientific study of cephalopod biology, which has been bolstered by the recent development of cephalopod tools, including genome and transcriptome sequences^14–18^, brain atlases^19–23^, cell atlases^24–27^, transient and multigenerational mutant animals^28, 29^, developmental staging series^30–32^, computational behavioral tools^33–36^, neural imaging using calcium dyes^37^, and electrophysiological recordings^38–40^.

Cephalopod research has traditionally been conducted in marine stations, relying on seasonal access to wild-caught cephalopods^41^. Over the past decades, however, multiple species have been successfully cultured through multiple generations in closed aquatic systems. Prominent species include the dwarf cuttlefish (*Ascarosepion bandense,* formerly *Sepia bandensis*)^30^, California two-spot octopus (*Octopus bimaculoides*)^42^, common cuttlefish (*Sepia officinalis*)^43^, Hawaiian bobtail squid (*Euprymna scolopes*)^44^, hummingbird bobtail squid (*Euprymna berryi*)^45^, and flamboyant cuttlefish (*Ascarosepion pfefferi*, formerly *Metasepia pfefferi*)^46^. These multi-generational cultures can provide year-round access to freshly fertilized eggs for microinjection, embryos of any stage for developmental studies, individuals at defined life stages for behavioral assays, and the opportunity to establish mutant or transgenic lines. Despite these advantages, stable and cost-effective cephalopod culture remains one of the greatest barriers to scientific progress: Cephalopods have high metabolic demands, are extremely sensitive to water quality, and have complex behaviors^47^. Thus, running a cephalopod facility requires specialist cephalopod expertise and considerable research expenses in equipment, personnel, and food costs.

Sex ratios in captive cephalopod populations can have a major impact on survival, behavior, and egg yield. For some cuttlefish species, mixed-sex groups can be maintained safely, but an excess of males can reduce egg production and increase aggression^48^. In many other cuttlefish species, mixed sex groups can become highly aggressive at sexual maturity, with males sometimes cannibalizing conspecifics unless housed in large holdings or kept in isolation^49^. By contrast, groups composed entirely of males can exhibit markedly lower aggression (unpublished observation). Sex-based management strategies can therefore be successful, but sex must be determined prior to sexual maturity. Anatomical determination of sex is theoretically possible: most mature male coleoid cephalopods possess a hectocotylus – a modified arm with fewer suckers, which is used to transfer spermatophores^13^ – but this modification can be subtle and difficult to detect. Some species exhibit sex-specific body sizes or social skin patterns, but such differences typically appear only after sexual maturity^13^, limiting their utility for early-stage sex identification. Beyond husbandry, accurate sex identification is essential for behavioral research. During social interactions, cuttlefish use a repertoire of innate skin patterns to communicate^6^, some of which are sex-specific. However, some species have been observed mimicking the opposite sex through skin patterning^50, 51^, making behavioral cues insufficient for reliable identification. A non-invasive method to determine sex in very young cephalopods could enable researchers to establish optimal sex ratios, which may in turn reduce aggression, increase reproductive output, and improve animal welfare. Additionally, an optimized sex ratio could reduce the number of animals required in a facility, potentially saving many thousands of dollars in yearly research costs and facilitating more rigorous experimental design.

**Figure 1.**
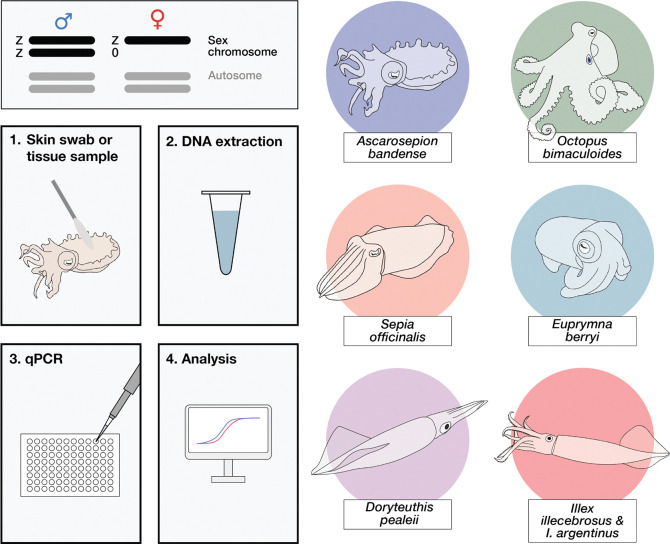
Overview of the protocol.

The recent chromosome-level assembly of the *Octopus bimaculoides* genome revealed the presence of a genetic sex-determination system in cephalopods, consisting of a Z0 sex karyotype in females and ZZ sex karyotype in males^52^. This sex system would, in theory, allow for an accurate diagnostic of sex from DNA alone. Here, we identified the orthologous Z chromosome in *A. bandense*, *S. officinalis*, *E. berryi*, and *D. pealeii*, optimized a method to non-invasively extract genomic DNA from live cephalopods as young as three hours post-hatching (8 mm body length; 5.3 mm mantle length *A. bandense*), and established a sensitive quantitative PCR (qPCR) protocol that can detect a two-fold difference in the number of Z sex chromosomes present in a DNA sample relative to autosomes, thereby differentiating males from females (Figure 1). Our method was 100% accurate, based on the confirmed post-mortem analysis of 81 *A. bandense*. Furthermore, we designed and validated qPCR primers for *O. bimaculoides, S. officinalis*, *E. berryi*, and *D. pealeii,* and identified Z-linked genes and primers in *Illex illecebrosus* using only low-coverage short-read data. The *I. illecebrosus* primers also accurately distinguished sex in the related *Illex argentinus*, demonstrating that this approach can be extended to species without a complete genome assembly. This method promises to facilitate the use of cephalopods in laboratory culture and research.

## Results

### Conserved synteny and homology reveal orthologous sex chromosomes in multiple coleoid species

**Figure 2.**
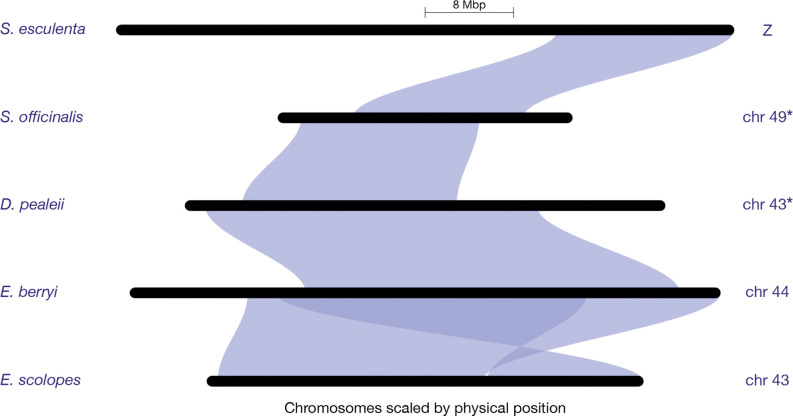
Conserved synteny relationships among putative Z chromosomes of *E. scolopes*, *E. berryi*, *D. pealeii*, *S. officinalis*, and *S. esculenta*. This riparian plot was generated from orthogroups with *E. scolopes* set as the reference species. The *S. officinalis* and *D. pealeii* chromosomes (marked with *) were inverted to improve visualization.

A cephalopod sex chromosome (Z) was previously identified within the California two-spot octopus (*O. bimaculoides*) genome^52^. To identify the putative Z chromosomes in other species, we examined conserved synteny across chromosome-scale genome assemblies of five coleoid cephalopods: *Sepia esculenta*^17^, *S. officinalis*^17^, *E. berryi*^24^, *E. scolopes*^15^, and *D. pealeii*^14^. In two species, *S. esculenta* and *E. scolopes*, the Z chromosomes were identified previously^52^. In three additional species, we identified the putative Z chromosomes: chromosome 43 (*D. pealeii*), chromosome 44 (*E. berryi*), and chromosome 49 (*S. officinalis*, which is consistent with other findings^53^) (Figure 2, Figure S1).

The current *A. bandense* genome assembly^54^ is not at chromosome level, so we could not include it in our conserved synteny analyses. Therefore, to identify the putative Z contigs in this species, we aligned the *A. bandense* genome scaffolds to the *E. scolopes* genome, and extracted contigs that aligned to the *E. scolopes* Z chromosome (chromosome 43).

### Quantitative PCR shows sex-specific dosage differences in octopus, cuttlefish, and squid species

**Figure 3.**
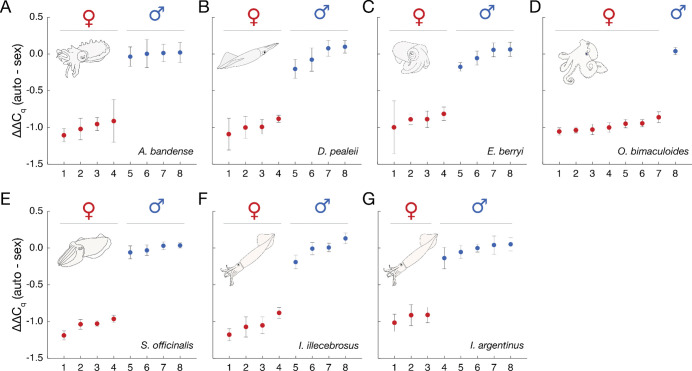
Quantitative PCR from the genomic DNA of seven squid, cuttlefish, and octopus species reveals sex-specific dosage differences. (A) *A. bandense.* (B) *D. pealeii.* (C) *E. berryi.* (D) *O. bimaculoides.* (E) *S. officinalis.* (F) *I. illecebrosus.* (G) *I. argentinus.* For each species, ΔΔCq values were calculated between autosomal and sex-linked loci across eight blinded, randomized tissue samples, normalized such that the median value of the greater-ΔΔCq cluster (male) was set to zero. Individuals are arranged along the x-axis in order of increasing ΔΔCq. Error bars represent the standard error of the mean of four technical replicates.

To test whether qPCR could be used to determine the sex of *A. bandense, O. bimaculoides, S. officinalis*, *E. berryi,* and *D. pealeii*, we designed primer sets targeting the Z chromosome (“sex” primers) and an autosome (“autosome” primers) using the Z chromosome sequences we identified (Figure 2). The goal was to measure whether the sex amplicon amplifies at equal the dosage (male) or half the dosage (female) of the autosome control. A two-fold difference in DNA template dosage amounts to a one-cycle difference in amplification; thus, we required primers with high specificity that generate a single melt curve representing a single product. Using stringent parameters (see Methods), we designed 24 primer sets for each species and tested them on DNA extracted from arm tissue from a confirmed phenotypic male and a confirmed phenotypic female cuttlefish. At least one primer set for each species yielded a ΔΔCq difference of 1 between the male and female DNA samples (see Methods), demonstrating sufficient sensitivity to detect a two-fold difference in sex chromosome dosage and generating a single amplicon (Table 1, Figure S2, Table S1). Using blinded, post-mortem tissue from eight individuals of known phenotypic sex per species, we performed qPCR and examined the resulting ΔΔCq values. In every case, the values segregated into two clusters corresponding to males (ΔΔCq = 0) and females (ΔΔCq = −1), yielding 100% correct assignments in each species (Figure 3A–E, 8/8, p = 0.0039, one-sided exact binomial test).

Many cephalopod species of biological and economic interest, such as the squid *Illex illecebrosus,* lack chromosome-level genome assemblies. To test whether short-read sequencing data are sufficient for primer design, we generated low-coverage whole genome sequencing data from *I. illecebrosus* and aligned them to the genome of the most closely related species with a reference genome, *D. pealeii*. Based on *D. pealeii* sequences, we designed a panel of 24 primer sets (12 sex, 12 autosome) and then refined the primer sequences to match *I. illecebrosus*-specific variants (Figure S3). From this panel, one primer set displayed the required performance (Table 1, Figure S2). We validated the primers on eight blinded *I. illecebrosus* individuals of known phenotypic sex, and achieved 100% sex attribution accuracy (Figure 3F, 8/8, p = 0.0039). Notably, the same primers also correctly identified sex in eight blinded samples of the related Argentine shortfin squid *Illex argentinus* (Figure 3G, 100%, 8/8, p = 0.0039). Thus, qPCR targets can be designed from low-coverage short-read sequencing data, and at least in some cases, can be functional for related species that lack extensive genome information.

### Quantitative PCR from non-invasive skin swabs successfully identifies sex

**Figure 4.**
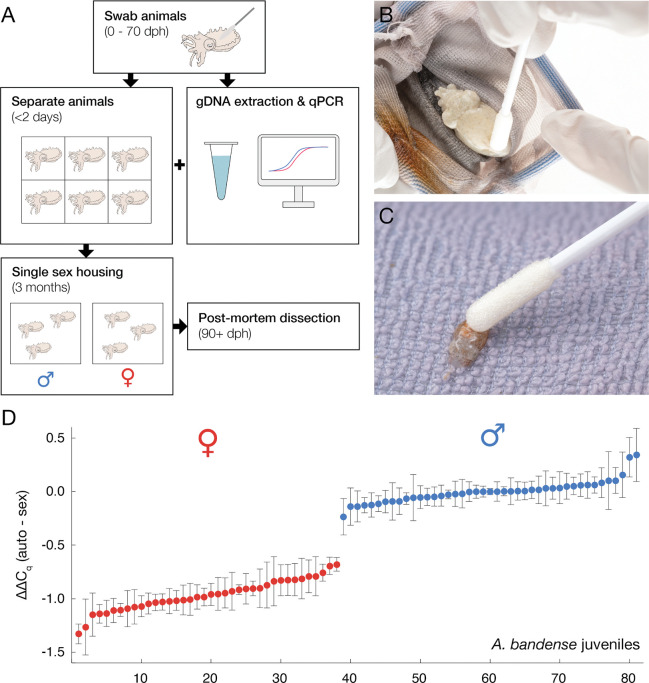
Quantitative PCR of genomic DNA from skin swabs of live juvenile *A. bandense* allows accurate sex determination. (A) Live animal sex genotyping workflow. Juvenile animals are swabbed and individually housed during the DNA extraction and qPCR. After obtaining qPCR results, animals are group housed by predicted genetic sex until confirmation of phenotypic sex by dissection. dph, days post-hatching. (B) A 10-week-old juvenile being swabbed for DNA extraction. (C) A 3-hour-old hatchling being swabbed for DNA extraction. (D) ΔΔCq values between autosomal and sex loci for 81 live juvenile *A. bandense* individuals, normalized (ΔΔCq = 0) to the median value of the male cluster. Individuals are ranked along the x-axis in order of increasing ∆∆Cq value. Error bars represent standard error of the mean. Predicted sex is indicated as red (female) or blue (male). All 81 predictions were confirmed by dissection.

A qPCR assay to determine animal sex would be most useful if it could be applied to live animals. We adapted a previously described squid genotyping protocol^29^ to swab the mantle skin of live, unanesthetized *A. bandense* and extract DNA from the swabs (100% survival, n = 81, Figure 4A–C). After running a qPCR with the validated *A. bandense* autosome and sex primers (Figure 3A), results yielded ΔΔCq values that clustered into two groups (median value distance of 0.97 and separation of 0.44 between the nearest data points from opposite groups) corresponding to putative males and females (Figure 4D). To validate these assignments, we performed post-mortem dissections after the animals reached sexual maturity, which revealed that the qPCR results were 100% accurate (81/81 correct, p = 4.14×10^−25^; Figure 4D). To find out how early in development animals could be swabbed and successfully sex genotyped, we swabbed eight hatchlings 3 hours post-hatching (5.3 mm mantle length; Figure 4C). These swabs yielded sufficient DNA for amplification by qPCR, the animals survived, and the genotyping results matched those obtained from genomic DNA from tissue extractions (Figure S4). Together, these results establish that a non-invasive skin swab can provide sufficient DNA for qPCR-based sex genotyping in *A. bandense*, even from newly hatched animals as small as ~5 mm mantle length.

## Discussion

This study demonstrates that a non-invasive skin swab combined with a qPCR assay can reliably determine the sex of coleoid cephalopods. The workflow is efficient: in our hands, swabbing 48 animals, extracting DNA, setting up and running a qPCR, and analyzing the results can all be completed within a single day (Figure 1). Importantly, this approach enables accurate sex determination across life stages – from hatchlings to adults – and across a range of cephalopod species, independent of genome quality or availability. Sex genotyping by qPCR can also be applied to embryos, and to post-mortem tissue, as has been demonstrated in the bigfin reef squid (*Sepioteuthis lessoniana*)^55^ and here (Figure 3).

This method has the potential to have a major impact on captive cephalopod population management. Populating breeding tanks with optimal sex ratios can maximize egg production while minimizing aggression. In turn, this can reduce the number of animals required in a culturing facility, reducing operational costs. The nature of this procedure allows for quick and efficient DNA collection, enabling accurate sex identification without the need for anesthesia or any invasive methods. These goals are in line with the principles of the 3Rs (Replacement, Reduction, and Refinement)56 and can help guide future welfare considerations for cephalopods.

Creating breeding groups for genetic diversity or genetically-modified lines can be both inefficient and high risk when using traditional sex identification, such as behavioral observations or hectocotylus identification. Our unpublished observations have shown that female dwarf cuttlefish that are paired with males after they reach sexual maturity will lay fewer eggs and be more susceptible to aggression or even cannibalism by males than those paired before sexual maturity. Furthermore, female cephalopods can carry sperm from different mates^57^ for days to months^58^ before successful fertilization, making any females that have previously lived with sexually mature males poor candidates for selective breeding. The ability to determine sex prior to sexual maturity permits longer cohabitation, which can reduce aggression and eliminate the risk of undesired sperm storage in females – a crucial resource in the pursuit of generating stable transgenic or mutant lineages.

Beyond husbandry applications, non-invasive sex genotyping can facilitate sex identification in juvenile cephalopods used in growth or physiological studies, identify environmentally mediated sex reversals^59^, and support fisheries management. For example, the northern and Argentine shortfin squids, *I. illecebrosus* and *I. argentinus,* are economically important species that are harvested in large volumes for seafood markets. The ability to non-destructively determine the sex of live animals, especially paralarvae or juveniles, could enable the assessment of sex ratios in wild populations during monitoring surveys, and assist fisheries managers and law enforcement in promoting sustainable harvesting through sex-specific catch limits. For such applications, portable rapid field assays would be more practical than laboratory-based qPCR, highlighting an important area for future development.

### Limitations of the study

There are two main limitations that impact the practicality of this method. First, qPCR instruments are expensive, and not all research laboratories or fisheries research vessels will have routine access to one. One solution could be to use commercial providers that offer fee-for-service qPCR, although costs and turnaround times may limit feasibility, especially given the need to house animals individually after swabbing. Second, primer design requires some amount of genetic sequence information for the target species. However, as our results show, low-coverage whole genome sequencing reads can be aligned to a closely-related species’ genome assembly to identify conserved loci in autosomes and sex chromosomes. As more cephalopod genomes become available, it may also become possible to design universal primers that function across species, expanding accessibility.

In summary, non-invasive sex genotyping by qPCR provides a versatile and accurate tool for research, enabling improved breeding, animal welfare, and resource management across cephalopods.

## Resource availability

### Lead contact

Requests for further information, resources, and reagents should be directed to and will be fulfilled by the lead contact, Tessa Montague (tessa.montague@columbia.edu).

### Materials availability

All items described here are commercially available.

## Materials and methods

### Ethics statement

The use of cephalopods in laboratory research is currently not regulated in the USA. However, Columbia University has established strict policies for the ethical use of cephalopods, including operational oversight from the Institutional Animal Care and Use Committee (IACUC). All of the cuttlefish used in this study were handled according to an approved IACUC protocol (AC-AABT8673), including the use of deep anesthesia and the minimization and prevention of suffering.

### Z chromosome identification in *D. pealeii*, *E. berryi*, *S. officinalis,* and *A. bandense*

To conduct synteny analyses, peptide and gtf files for *D. pealeii* and *E. scolopes* were downloaded from their associated publications^14, 15^ while an unpublished assembly associated with the *E. berryi* cell atlas^24^ was used for *E. berryi* (see Table S1 for assembly information). An annotation was previously generated for *S. esculenta* by a gene annotation liftover from *S. pharaonis*^52^. At the time of our analyses, an annotation for the genome assembly of *S. officinalis* was not available. Therefore, we generated an annotation for *S. officinalis* by lifting over the genome annotation from *S. pharaonis* using Liftoff^60^ with default settings. Synteny analyses between the chromosomes of *S. esculenta*, *S. officinalis*, *E. berryi*, *E. scolopes*, and *D. pealeii* were performed using the R package GENESPACE v.1.2.3^61^. In all synteny maps, *E. scolopes* was used as the reference species. In riparian plots and dotplots, the chromosomes are scaled by physical position (useOrder = False parameter in GENESPACE). The *A. bandense* genome assembly^54^ and associated transcriptome was pulled from cuttlebase.org/downloads. Since the *A. bandense* genome was not a chromosome level assembly, we could not use gene synteny to identify the putative Z chromosome. Instead, we used minimap2^62^ to align *A. bandense* to *E. scolopes* (with parameters -cx asm20) to identify potential Z contigs.

### Generation of *I. illecebrosus* short-read sequences and alignment to *D. pealeii* genome

To identify autosomal and sex-linked primers in *I. illecebrosus*, a set of primers was first designed for *D. pealeii* using orthologous genes identified by GENESPACE targeting chromosome Dpe01 (“autosome” primer) and chromosome Dpe43 (“sex” primer). Next, short-read sequencing data was generated from 20 *I. illecebrosus* individuals (10 males and 10 females, as determined by gonad morphology). Specifically, squid DNA was extracted using a DNeasy Blood & Tissue Kit (Qiagen) following the manufacturer’s instructions, and DNA extracts were purified and cleaned from short DNA fragments using TotalPure NGS Mag-Bind Beads (Omega Bio-tek). Low-coverage Whole Genome Sequencing Illumina libraries were prepared using the miniaturized Nextera DNA Library Preparation Kit with an Echo liquid handler and sequenced by the Genomics and Cell Characterization Core Facility (GC3F) at the University of Oregon using an Illumina NovaSeq 6000. Raw sequencing data was processed using grenepipe^63^ by aligning to the *D. pealeii* reference genome (GCA_023376005.1). The resulting BAM files were used to make a consensus using the SAMtools consensus tool^64^.

### qPCR primer design

For all species, 12 primer pairs were designed against autosomal genomic DNA sequences and 12 primer pairs against known or presumed sex chromosomal genomic DNA sequences. The targeted chromosome sequences are listed in Table S1. Primer design was performed as described below, and the final primer sequences, binding sites, and amplicon sequences are indicated in Table S1.

#### A. bandense, O. bimaculoides, S. officinalis, E. berryi, D. pealeii:

Primers were designed using Geneious Prime 2025.2.2 (https://www.geneious.com) with a modified Primer3 (2.3.7,^65^) incorporating the following parameters: optimal primer size of 20 bp, optimal Tm of 59°C, optimal GC content of 50%, product size between 100–200 bp, GC clamp of 1, max poly-X of 3, and max dimer Tm 30°C. Primers were further screened for off-targets by a BLAST search against the appropriate reference genomes, and for secondary structures and self-complimentarity using IDT OligoAnalyzer (https://www.idtdna.com/calc/analyzer).

#### I. argentinus, I. illecebrosus:

Primer pairs were generated using the ceph_primer_pipeline.sh (available at www.github.com/stsmall/ceph_primer_pipeline). This command-line script uses Primer3 to design primers (optimal primer size of 20 bp, optimal Tm of 60°C, optimal GC content of 50%, product size between 150–300 bp, and max poly-X of 5) and MFEprimer^66^ to check primer specificity against a reference genome. Using a GFF file and reference genome in FASTA format, this script uses BEDTools^67^ to extract user selected attributes (e.g. exons) from a given chromosome and builds a FASTA file for input into Primer3. The Primer3 default config file was modified to return only a single set of primers per FASTA entry, and the final primer sets are exported as a CSV file.

Primers were first tested against genomic DNA extracted from confirmed male and female individuals (see Genomic DNA extraction and Quantitative PCR sections below), as well as no-template controls. Primer pairs which gave a single, template-dependent melt curve were analyzed by 2% agarose gel electrophoresis and sequenced to confirm the generation of a single PCR product, which was validated against the reference sequence. The sequencing of PCR products was performed by Plasmidsaurus using Oxford Nanopore Technology with custom analysis and annotation (Premium PCR Sequencing service). For each species, a single autosome-targeting primer pair and a single sex chromosome-targeting primer pair were chosen for further sex genotyping analysis.

### Genomic DNA extraction from cephalopod frozen tissue

Frozen arm tips from *O. bimaculoides, S. officinalis, E. berryi,* and *D. pealeii* were obtained as flash frozen samples, while *I. illecebrosus* and *I. argentinus* samples were obtained as mantle fragments or arm tips preserved in 95% ethanol. All samples were temporarily stored at −80°C until DNA extraction. To extract genomic DNA, ~3 mm pieces of tissue were placed in 1.5 ml Safe-Lock tubes, and processed using the Monarch Spin gDNA Extraction Kit. Briefly, 200 μl of Tissue Lysis Buffer and 10 μl of 20 mg/ml Proteinase K were added to each tube, and the samples were incubated at 56°C in a ThermoMixer (Eppendorf) shaking at 1400 rpm for 1–2 hours until fully digested. In some cases, the lysate turned pink or purple, due to chromatophore pigments. Following digestion, 400 μl of Binding Buffer was added, samples were pulse vortexed for 5–10 seconds, and the lysates were transferred to gDNA Purification Columns. Columns were centrifuged at 1,000 x *g* for 3 minutes followed by >12,000 x *g* for 1 minute, washed twice with 500 μl of gDNA Wash Buffer, and eluted with 60 μl of pre-warmed (60°C) gDNA Elution Buffer after a 5 minute incubation at room temperature. Eluted DNA concentrations ranged from ~50 to 350 ng/μl. All DNA was diluted 20-fold (no further normalization required) and transferred into 8-well strips for subsequent qPCR.

### Quantitative PCR

qPCR reactions were performed on a QuantStudio 5 Real-Time PCR System (Applied Biosystems) using 2X SYBR Green qPCR Master Mix (APExBIO) with ROX reference dye (APExBIO). Reactions were prepared as technical repeats in quadruplicate (7 μL total volume, 0.25 μM forward primer, 0.25 μM reverse primer, 2 μL template DNA). Thermocycling conditions were as follows: initial denaturation (95°C for 2 min), amplification (95°C for 15 s, 60°C for 45 s, for a total of 45 cycles), melt curve analysis (95°C for 15 s, 60°C for 1 min, followed by an increase to 95°C at a rate of 0.06°C/s).

### Quantitative PCR analysis

Cq values, mean values, and standard error of the mean were automatically determined using Design & Analysis Software v2.2.0 (Applied Biosystems). Product identity was validated by melt curve comparison with sequence-verified standards (see qPCR primer design above). Outliers within a replicate group (Cq values differing by > 1 cycle from the mean) were discarded prior to analysis. 0394Cq values were calculated as Cq (autosome) minus Cq (sex chromosome) and were confirmed to cluster into two groups. ΔCq values were calculated by subtracting the median value of the male (i.e., the greater ΔCq) cluster, or the value of a male standard. Reported standard error of the mean (SEM) values for each individual represent the sum of its SEM values from autosome and sex replicate groups. Data visualization and analysis were performed in Design & Analysis Software, MATLAB R2024b, and Microsoft Excel. For each species, a one-sided exact binomial test was used to determine if the observed rate of correct predictions performed better than chance (50%).

### Skin swabs and genomic DNA extraction

Dwarf cuttlefish were carefully caught with a soft net and positioned dorsal side up without anesthesia. A sterile foam swab was pre-soaked in tank water and gently stroked across the dorsal mantle surface from left to right, avoiding the anterior tip of the mantle (the most delicate region of the skin). The animal was then gently rotated ventral side up, and the other side of the swab was used to collect cells from the ventral surface with slightly greater pressure. Each animal was placed in an individual tank to keep track of its identity, and the swabs were placed face down into 1.5 ml Safe-Lock tubes preloaded with 200 μl Tissue Lysis Buffer and 3 ul of 20 mg/ml Proteinase K. After all samples were collected, swab shafts were trimmed to allow tube closure, and samples were incubated at 56°C in a ThermoMixer (Eppendorf) shaking at 1400 rpm for 1 hour. The shortened shaft of each swab was then pressed and dragged against the side of the tube to recover liquid from the foam tip. Next, 400 μl of Binding Buffer was added, the samples were pulse vortexed for 5–10 seconds, and the lysate was transferred to gDNA Purification Columns. Columns were centrifuged at 1,000 x *g* for 3 minutes followed by > 12,000 x *g* for 1 minute, washed twice with 500 μl of gDNA Wash Buffer, and eluted after a 5 minute incubation with 60 ul of pre-warmed (60°C) gDNA Elution Buffer. Typical yields from an animal of 10–20 mm mantle length ranged from 5–25 ng/μl (225 ng–1.1 μg total). DNA was transferred into 8-well strips for qPCR, with no further normalization of concentration required.

## Supplementary Material

Supplement 1

Supplement 2

Supplement 3

Supplement 4

## Figures and Tables

**Table 1.** qPCR primer sequences for cuttlefish, octopus and squid species. For additional primer details, see Table S1.

Organism	Primer name	Primer sequence (5’→3’)
***Ascarosepion bandense***	Abandense_qPCR_auto_F	GGTGTTGTTCGCTCAGTTATC
	Abandense_qPCR_auto_R	GTGTTCATGTCGCCATCTTATC
	Abandense_qPCR_sex_F	GGCCGTCCTCTACTTGTAATG
	Abandense_qPCR_sex_R	AGTAGCTGTGTGGTTGAGAAG
***Octopus bimaculoides***	Obimaculoides_qPCR_auto_F	TTGTTTGGACCTTGGGCTTATAG
	Obimaculoides_qPCR_auto_R	CTGTCATGAACCCTGGTGTATTC
	Obimaculoides_qPCR_sex_F	CCTCACCACTGGATGCAATTAAG
	Obimaculoides_qPCR_sex_R	GCCAATCCGTCCAACCTATAC
***Sepia officinalis***	Sofficinalis_qPCR_auto_F	TTTGCCACTGTGTCCCTTTATAC
	Sofficinalis_qPCR_auto_R	ACACACACAGGCTGCTTATTG
	Sofficinalis_qPCR_sex_F	TTTCAACCCATCTGCGTCTATAG
	Sofficinalis_qPCR_sex_R	ACTCCTCTCGTTGCATGATTAC
***Euprymna berryi***	Eberryi_qPCR_auto_F	CTTTCGCCACGCCTGATATAC
	Eberryi_qPCR_auto_R	CAGCAGCTTCTTTCCCAGATAAG
	Eberryi_qPCR_sex_F	CTGCCCAGCGAATTGTTTATTG
	Eberryi_qPCR_sex_R	TCCGGCGTCTAGGGATTTAG
***Doryteuthis pealeii***	Dpealeii_qPCR_auto_F	CACTTCAGCCCGATGGAATAAG
	Dpealeii_qPCR_auto_R	CTTTGTAAATGCCGCACCTATATC
	Dpealeii_qPCR_sex_F	GGAGTCTGAGGTCCGAGATATAG
	Dpealeii_qPCR_sex_R	GCCGAGACCACAAACAATAAC
***Illex illecebrosus & Illex argentinus***	Illex_qPCR_auto_F	AAAACTCCCGACGTCTTGAA
	Illex_qPCR_auto_R	GGCCATCCTGGTAGACAAGA
	Illex_qPCR_sex_F	AATCACCCCAACCAGATGAA
	Illex_qPCR_sex_R	CTCCTGGACCTGGAATGAAA

**Key Resources Table:** 

REAGENT or RESOURCE	SOURCE	IDENTIFIER
**Chemicals, peptides, and recombinant proteins**		
2X SYBR Green qPCR Master Mix	APExBIO	K1070
Mag-Bind TotalPure NGS	Omega Bio-tek	M1378-00
**Biological samples**		
*Euprymna berryi* tissue	Marine Biological Laboratory	N/A
*Octopus bimaculoides* tissue	Marine Biological Laboratory	N/A
*Doryteuthis pealeii* tissue	Marine Biological Laboratory	N/A
*Sepia officinalis* tissue	Marine Biological Laboratory	N/A
*Illex illecebrosus* tissue	NOAA Northeast Fisheries Science Center	N/A
*Illex argentinus* tissue	Falkland Island Fisheries Department	N/A
**Critical commercial assays**		
Monarch Spin gDNA Extraction Kit	New England Biolabs	T3010L
DNeasy Blood & Tissue Kit	Qiagen	69504
**Experimental models: Organisms/strains**		
*Ascarosepion bandense*	This paper	N/A
**Oligonucleotides**		
See Table 1.		
**Software and algorithms**		
Primer3	Koressaar et al., 2018^65^	https://primer3.org
Geneious Prime 2025.2.2	N/A	https://www.geneious.com
Design & Analysis Software v2.2.0	Applied Biosystems	
GENESPACE v1.2.3	Lovell et al., 2022^61^	https://github.com/itlovell/GENESPACE
Liftoff v1.6.3	Shumate and Salzberg, 2021^60^	https://github.com/agshumate/Liftoff
Minimap2-2.30 (r1287)	Li, 2021^62^	https://github.com/lh3/minimap2
Grenepipe v0.15.0	Czech and Exposito-Alonso, 2022^63^	https://github.com/moiexpositoalonsolab/grenepipe
SAMTools v1.22.1	Danecek et al., 2021^64^	https://github.com/samtools/samtools
ceph_primer_pipeline.sh	This paper	www.github.com/stsmall/ceph_primer_pipeline
BEDTools v2.31.1	Quinlan and Hall, 2010^67^	https://github.com/arq5x/bedtools2
MFEPrimer v4.2.3	Wang et al., 2019^66^	https://github.com/quwubin/MFEprimer-3.0
Other		
6" Sterile Standard Foam Swab w/ Polystyrene Handle	Puritan	25-1506 1PF 100
1.5 ml Safe-Lock tubes	Eppendorf	0030123611
SnapStrip 8-Strip Standard PCR Tubes with Individually Attached Flat Caps	GeneMate	490003-706
MicroAmp EnduraPlate Optical 384-Well Clear Reaction Plates	Applied Biosystems	4483285
MicroAmp Optical Adhesive Film	Applied Biosystems	4311971
Nuclease-free water	Ambion	AM9930
QuantStudio^™^ 5 Real-Time PCR System, 384- well	Applied Biosystems	A28140

## Highlights

- The Z sex chromosome was identified in multiple cuttlefish, squid, and octopus species.
- A sensitive quantitative PCR assay can genotype ZZ/Z0 sex in each species.
- Low-coverage short-read sequencing data is sufficient to design effective primers.
- qPCR on non-invasive skin swabs enables genotyping of living animals.
