# Supplementary material for: A non-invasive method to genotype cephalopod sex by quantitative PCR
Source: bioRxiv. 2025 Oct 29:2025.10.28.685099. Preprint. [Version 1] doi: 10.1101/2025.10.28.685099 (PMC12636484; doi:10.1101/2025.10.28.685099)
Supplement: Supplement 1 [file media-1.pdf]

Supplemental Information

A non-invasive method to genotype cephalopod sex by quantitative PCR

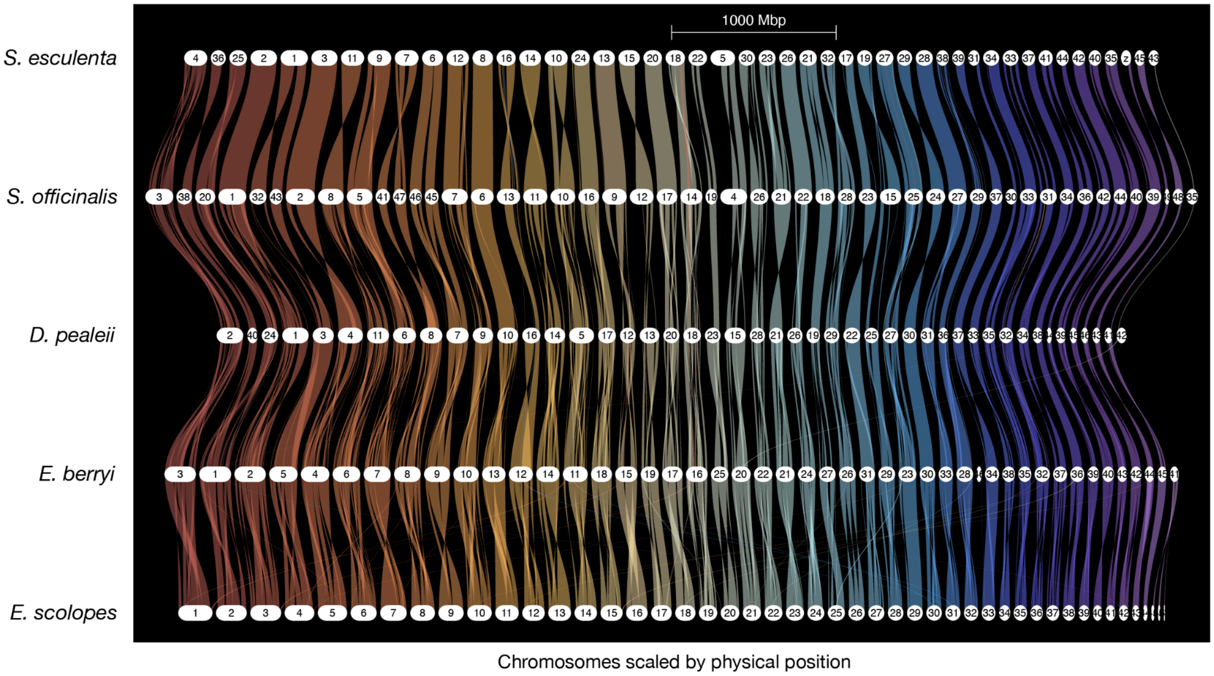

Figure S1. Conserved synteny relationships among chromosomes of *E. scolopes, E. berryi, D. pealeii, S. officinalis*, and *S. esculenta*. This riparian plot was generated from orthogroups with *E. scolopes* set as the reference species.
