# Supplementary material for: A non-invasive method to genotype cephalopod sex by quantitative PCR
Source: bioRxiv. 2025 Oct 29:2025.10.28.685099. Preprint. [Version 1] doi: 10.1101/2025.10.28.685099 (PMC12636484; doi:10.1101/2025.10.28.685099)
Supplement: Supplement 2 [file media-2.pdf]

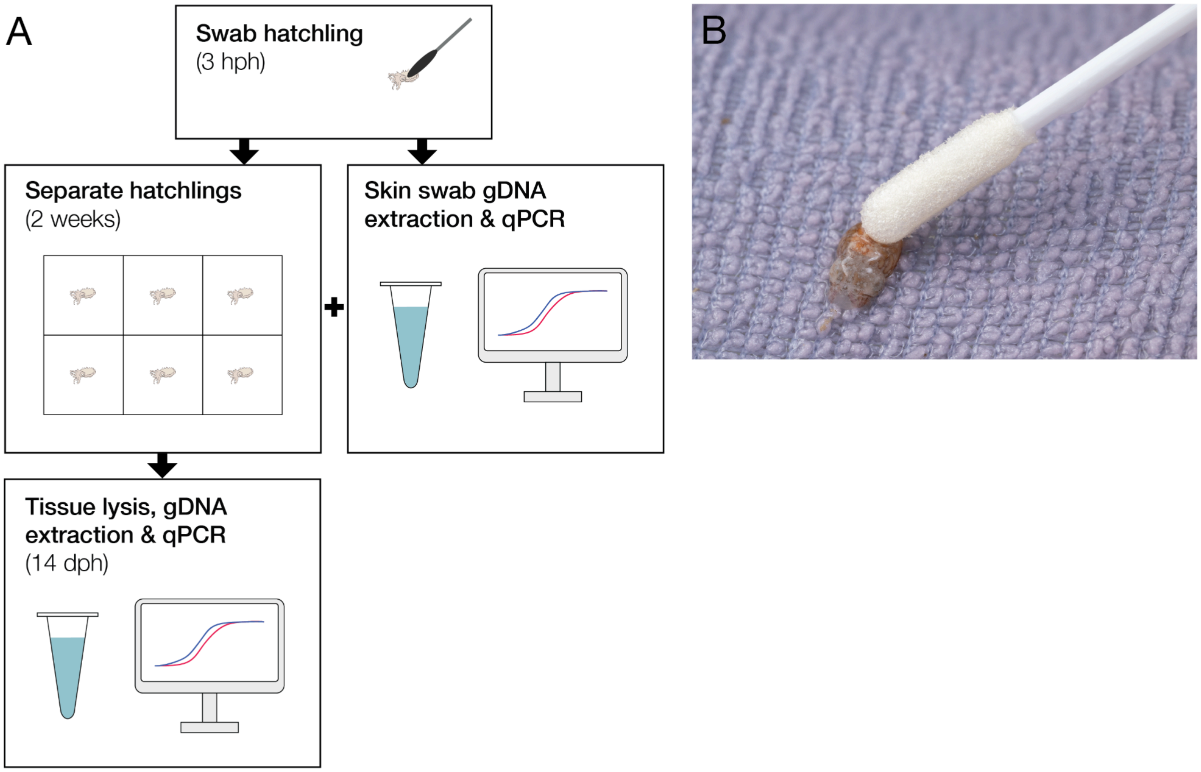

Figure S4. Swabbing 3-hour-old hatchlings yields sufficient DNA for qPCR. (A) Hatchlings were swabbed within three hours post-hatching (3 hph), gDNA was extracted from the skin swab and used for qPCR-based sex prediction. After swabbing, the hatchlings were housed separately for two weeks to ensure their survival. At 14 days post-hatching (14 dph) the hatchlings were euthanized, gDNA extracted from tissue, and used as input for qPCR. The predictions from both samples were compared for consistency.
