# Supplementary material for: A non-invasive method to genotype cephalopod sex by quantitative PCR
Source: bioRxiv. 2025 Oct 29:2025.10.28.685099. Preprint. [Version 1] doi: 10.1101/2025.10.28.685099 (PMC12636484; doi:10.1101/2025.10.28.685099)
Supplement: Supplement 3 [file media-3.pdf]

A

Autosome

```
                 Illex.1:    1-  100
I. illecebrosus  AAAACTCCCGACGTCTTGAAGGACAAATTGAGGGAAGCAATGAGAACAGGAGACAACGACGCTCTGGAAGCTGCTCTTGACGAGTGTGTTGCCGCTGGCA
                 ||||||||||||||||||||||||||| |||||||||| ||||||| ||||||||| |||||||||||| || |||||| || ||| | ||||||||||
D. pealeii       AAAACTCCCGACGTCTTGAAGGACAAACTGAGGGAAGCCATGAGAAGAGGAGACAAGGACGCTCTGGAAGATGTTCTTGATGAATGTATCGCCGCTGGCA
                 Dpe01:65236255-65236100:    1-  100

                 Illex.1:  101-  160
I. illecebrosus  TGCCAGAACTGGACGCCGATATTCACAGAGCAAGAAAAGCTCTTGTCTACCAGGATGGCC
                 |||||||| | ||||| |||||||||||||||||||||||||||| | ||||||||||||
D. pealeii       TGCCAGAATTAGACGCTGATATTCACAGAGCAAGAAAAGCTCTTGACGACCAGGATGGCC
                 Dpe01:65236255-65236100:  101-  160
```

B

Sex chromosome

```
                 Illex.Z:1-100
I. illecebrosus  AATCACCCCAACCAGATGAATCTGAATTAGCAGCTGCAGCAGACTTAGATGCTTCGGTTGTGTTTTTTGATGCTATTTCAGATGATGTTAAAGGATTGTT
                 |||| ||||| |||||||||||||| ||||||||||||||||||||||||||||| |||||||| |||||  ||||| ||||||||  || |||||||||
D. pealeii       AATCCCCCCAGCCAGATGAATCTGAGTTAGCAGCTGCAGCAGACTTAGATGCTTCAGTTGTGTTATTTGAATCTATTGCAGATGATAGTATAGGATTGTT
                 Dpe43:25792259-25792529:1-100

                 Illex.Z:101-200
I. illecebrosus  CTTGCTGCCCGGAGCAGCTCCAATGGCAGATGTGTCTGCAGAAGAGGAATTTAGATCACCAAGAATATCCGACAAAGATGCAGATGAAGATGCGGCTGGG
                 ||| ||||| |||||||||||||||||||||||||| |||| |  || ||| |  || ||||  | |||   |||||| ||||||||||||||||||||
D. pealeii       CTTACTGCCTGGAGCAGCTCCAATGGCAGATGTGTCCGCAG-A-TGG-ATTCATCTCGCCAAACAAATCTACCAAAGAGGCAGATGAAGATGCGGCTGGA
                 Dpe43:25792259-25792529:101-197

                 Illex.Z:201-273
I. illecebrosus  TTTGCTGAGGGCTGGCTCCCAACAGTTGGGCTAGATCCAGGAGGGGCAAGTATTTTCATTCCAGGTCCAGGAG
                 ||||||||| ||||| |||||||||||||||||||||||||||| ||||||||||||||| ||||||||||||
D. pealeii       TTTGCTGAGTGCTGGATCCCAACAGTTGGGCTAGATCCAGGAGGTGCAAGTATTTTCATTGCAGGTCCAGGAG
                 Dpe43:25792259-25792529:198-270
```

Figure S3. Amplicons used for sex genotyping in *I. illecebrosus*, showing the alignment of *I. illecebrosus* reads to the *D. pealeii* genome. (A) Autosomal amplicon (aligned to *D. pealeii* chromosome 1). Red line marks the *I. illecebrosus* primers. The forward primer has no mismatches, while the reverse primer has mismatches at position 13 (C>A) and position 15 (T>A). (B) Sex (Z) chromosome amplicon (aligned to *D. pealeii* chromosome 43). Blue line marks the *I. illecebrosus* primers. The forward primer has two mismatches (position 5 C>A and position 11 G>A), while the reverse primer has one mismatch at position 13 (C>G).
