# Supplementary material for: A non-invasive method to genotype cephalopod sex by quantitative PCR
Source: bioRxiv. 2025 Oct 29:2025.10.28.685099. Preprint. [Version 1] doi: 10.1101/2025.10.28.685099 (PMC12636484; doi:10.1101/2025.10.28.685099)
Supplement: Supplement 4 [file media-4.pdf]

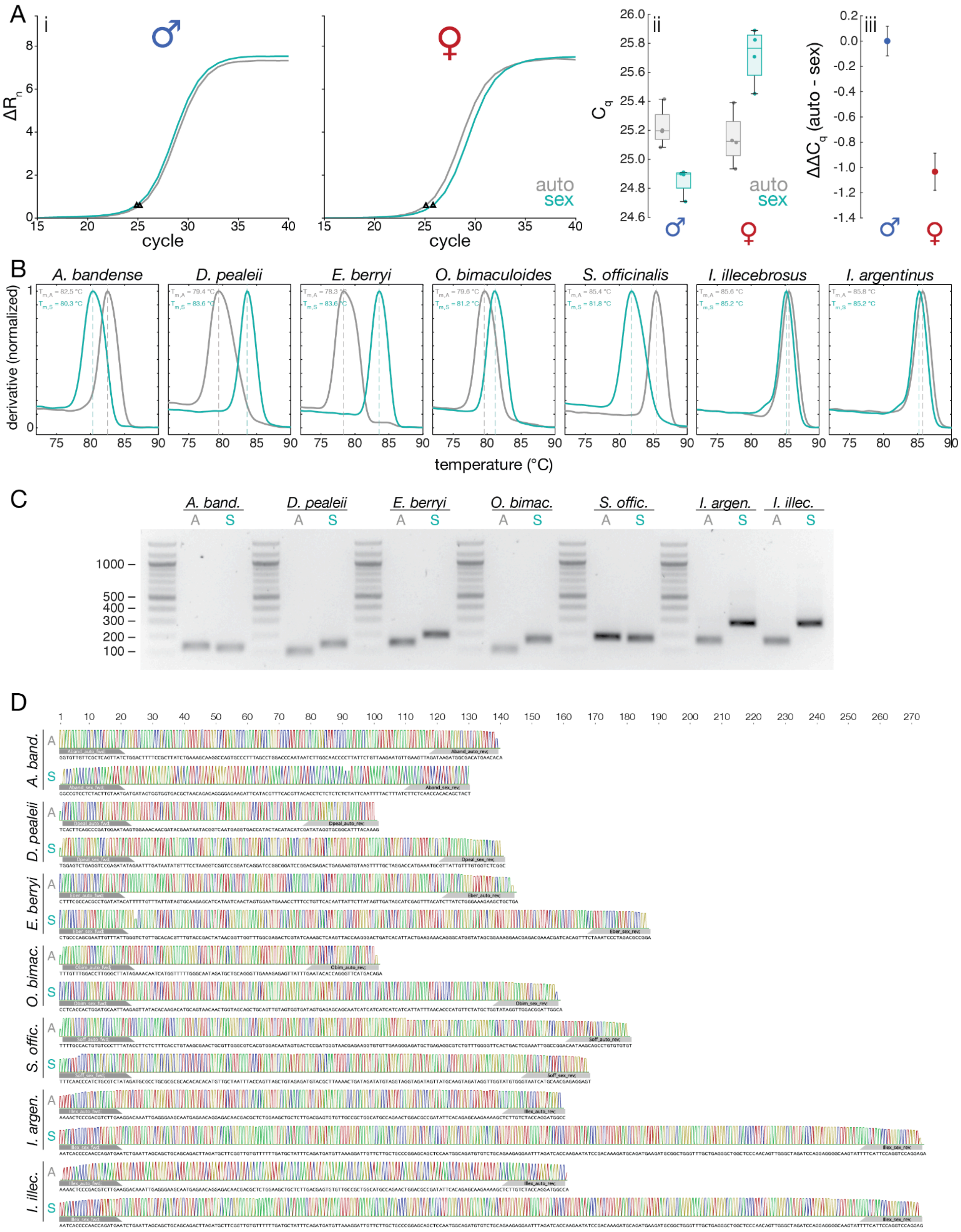

Figure S2. Validation of primer pairs used for quantitative PCR against autosome and sex chromosome loci of seven cephalopod species. (A) determination of $\Delta\Delta C_q$ values for a single male and a single female

standard of *A. bandense*: (i) amplification curves of male and female standards using autosome- and sex chromosome-targeting primer pairs (specified in Table S1). Triangles (△) represent C_q values as determined by Design & Analysis Software. For visual clarity, only a single technical replicate is shown for each; (ii) C_q values from four technical replicates of each reaction. Boxplots show the interquartile range with median line; whiskers indicate minimum and maximum values. (iii) Normalized $\Delta\Delta C_q$ values determined from four technical replicates (see Quantitative PCR analysis in Methods). Error bars indicate the summation of standard error of the mean values of $C_{q,auto}$ and $C_{q,sex}$. (B) Melt curve analysis of all primer pairs used in this study (Table S1). Melting temperature (T_m) as determined by Design & Analysis Software is indicated by a dashed line. (C) Agarose gel electrophoresis of qPCR amplicons. (D) Raw signal trace from nanopore sequencing of qPCR amplicons, with primer binding sites indicated.
